# Differential Thermal Inactivation Enables Simultaneous Quantitation of Ricin and Abrin

**DOI:** 10.3390/toxins18050233

**Published:** 2026-05-19

**Authors:** Woo-Hyeon Jeong

**Affiliations:** 3rd R&D Institute, 5th Directorate, Agency for Defense Development, Daejeon 34186, Republic of Korea; jwh3114@add.re.kr; Tel.: +82-10-4508-0329

**Keywords:** ricin, abrin, thermal stability, coexisting sample

## Abstract

Ricin and abrin are highly lethal Type II ribosome-inactivating proteins. They depurinate the same site of the 28S rRNA to inhibit protein synthesis. Consequently, standard molecular-level activity assays used to detect the toxic activity of ricin or abrin do not distinguish between the two in mixed samples without prior physical separation or specially designed substrates. This study proposes a novel, cost-effective method to separately and simultaneously quantify the activities of ricin and abrin in mixtures by exploiting their distinct thermal stabilities. Thermal inactivation was used to demonstrate that heating samples at 80 °C for 5 min maximized the difference in their activities; while ricin retained most of its activity, abrin activity dropped to 20% after thermal treatment. This thermal treatment yielded 4 standard curves—ricin or abrin, thermally treated or not treated—in the 0.3 to 50 µg/mL range. By applying Cramer’s rule, the individual concentrations of active ricin and abrin in mixed samples were successfully calculated. However, this method should be used with a method detecting presence of ricin/abrin, to avoid unexpected reactivity due to contaminating RIPs.

## 1. Introduction

Ricin, derived from the castor bean (*Ricinus communis* L.), and abrin, derived from the rosary pea (*Abrus precatorius* L.), are among the most potent and lethal biotoxins [1]. Both toxins are Type II ribosome-inactivating proteins, consisting of an A-chain and a B-chain linked by a disulfide bond. The B-chain facilitates cell entry by binding to galactose/*N*-acetylgalactosamine residues on the cell surface, whereas the A-chain exerts its toxicity by functioning as a highly specific RNA *N*-glycosidase [2]. Specifically, it depurinates the conserved adenine residue (A4324 in rat 28S rRNA) within the sarcin/ricin loop of the large ribosomal subunit [2]. This irreversible modification prevents binding by elongation factors, halting protein synthesis and leading to cell death via apoptosis [3,4]. Due to its extreme toxicity, ease of production, and historical use in espionage and warfare, ricin is strictly regulated as a Schedule 1 chemical agent by the Organization for the Prohibition of Chemical Weapons [5].

To mitigate the risks posed by these toxins, various analytical methods have been developed to assess their biological activity [6]. Traditionally, cytotoxicity assays using Vero cells have been the gold standard because they capture the entire intoxication process, including cell binding, internalization, and translocation [7,8]. However, cell-based assays are time-consuming and must be performed in specialized biosafety facilities. Alternatively, molecular-level assays focusing on the *N*-glycosidase activity of the A-chain have gained traction. For instance, adenine depurination assays utilize liquid chromatography–tandem mass spectrometry (LC-MS/MS) to monitor the release of adenine from designed substrates, such as specific ssDNA or ssRNA sequences that mimic SRL [9,10,11]. Cell-free translation systems, such as rabbit reticulocyte lysate assays, offer a faster means of measuring inhibitory activity without the need for complex cell culture [12,13].

A significant challenge in biothreat detection arises when ricin and abrin coexist in a single sample. Since both toxins share the same molecular mechanism [14], existing activity assays cannot distinguish them in a mixed sample. Recent efforts have sought to address this lack of selectivity. Dong et al. used sets of specially designed depurination substrates and dedicated depurination conditions for each toxin to differentiate between the toxins’ activities [15]. Although innovative, this approach is inherently limited to depurination-based assays and may not be applicable to other detection platforms. Conversely, Worbs et al. proposed an immunological approach that uses immunoprecipitation (IP) to physically separate and enrich for the target toxin prior to activity measurement [16,17], and Drinkard et al. developed an affinity enrichment technique using asialofetuin to physically separate type II RIPs from matrices [18]. This method significantly enhances sensitivity and provides the desired selectivity; however, it requires high-quality, toxin-specific antibodies, which are often costly or difficult to obtain.

The thermal stability of proteins is governed by their unique folding structures and amino acid sequences. Despite their functional similarities, ricin and abrin exhibit different thermodynamic profiles and temperatures for denaturation [19,20]. Exploiting these intrinsic physical differences offers a potential pathway for discrimination that does not rely on biological reagents like antibodies or specialized synthetic substrates.

In this research, I propose a novel method to simultaneously and separately quantify the activities of ricin and abrin in mixed samples using differential thermal inactivation, without the need for specific substrates or prior physical separation. The purpose of this study is to provide a simple, cost-effective method that can be applied broadly. By carefully controlling the heating parameters, this study demonstrated that one toxin can be selectively inactivated while the other retains its enzymatic potency. This method could provide a “physical filter” not confined to a specific assay type—whether it be adenine depurination with LC-MS/MS, cell-free translation, or ELISA-based functional assays. By eliminating the need for sophisticated prerequisites or expensive antibodies, this approach offers a robust and versatile solution for forensic analysis and environmental monitoring of dual-toxin threats.

## 2. Results

### 2.1. Comparing the Thermal Stability of Ricin and Abrin

**Figure 1:**
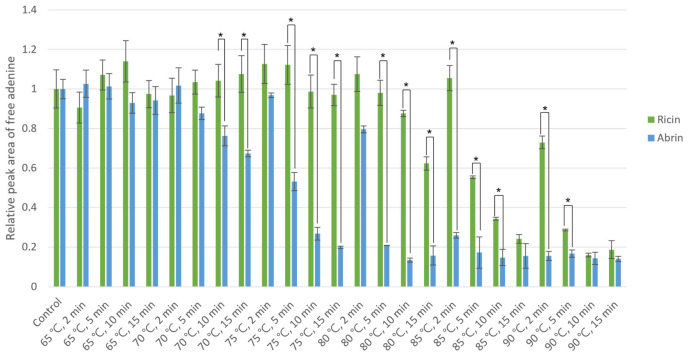
Depurinating activity of ricin (green) and abrin (blue) after thermal treatment. Toxin concentrations were 5 µg/mL. Results are the average of 3 independent assays. Error bars represent the SD of the averages. Conditions with asterisks indicate statistical significance at *p* < 0.005.

**Figure 2:**
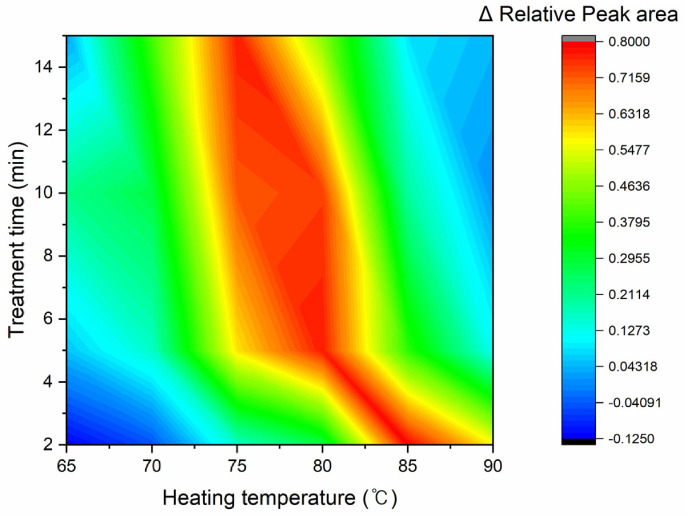
Contour map visualizing activity differences between two toxins after thermal inactivation. Blue and red represent identical activity or maximum difference in activity between two toxins, respectively.

Previous studies have described the thermal stability of ricin and abrin [19,20,21,22]. However, few studies have compared their thermal stabilities. This study attempted to identify conditions that maximize the difference between the two toxins’ activity. Ricin and abrin were individually dissolved in deionized water and heated to 65 °C, 70 °C, 75 °C, 80 °C, 85 °C, or 90 °C for 2, 5, 10, or 15 min to determine changes in their enzymatic activity. Buffers and substrates for adenine depurination were added to these thermally treated samples, and the resulting free adenine was quantified by LC-ESI-MS/MS (SRM). Figure 1 describes the relative depurinating activity of each treated toxin compared with the untreated controls. The activities of both toxins become statistically distinct when treated at temperatures >75 °C for 10 min. As shown in Figure 1, ricin retained much of its depurinating activity at 80 °C, whereas abrin lost approximately 80% of its activity. Meanwhile, both toxins retain most of their activity up to 70 °C, consistent with previous studies on their thermal stability [23,24], although this study focuses only on A-chain activity. The optimal condition was identified by normalizing the adenine peak areas to untreated controls and calculating the maximum difference in activity between the two toxins. In Figure 2, red indicates a greater difference between the normalized activities. The difference is maximized from 75 °C to 80 °C; ricin loses activity at temperatures >85 °C.

**Figure 3:**
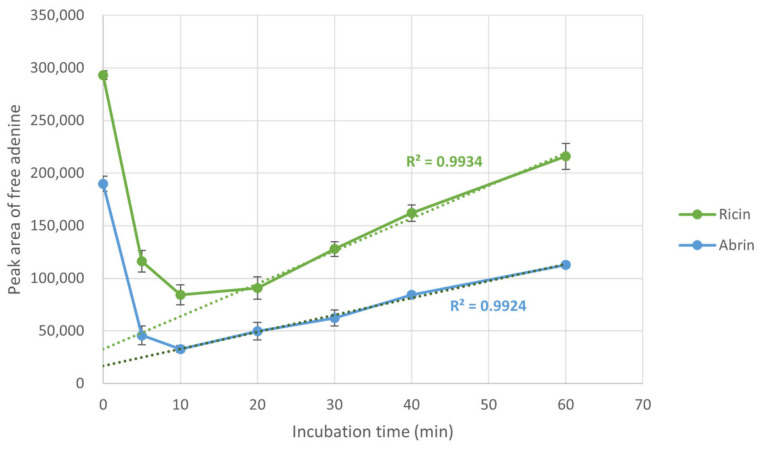
Extended thermal treatment test for ricin (green) and abrin (blue) in the presence of substrate ssDNA to assess spontaneous release of free adenine from the substrate. Three independent assays were performed and averaged. Dotted line represents trending line for each toxin. Error bars represent SD. Trending lines with corresponding colors represent the correlation between the incubation time and peak area over 20 min.

Since the experiment uses ssDNA as the substrate, its intrinsic tendency to release free adenine upon thermal treatment should be considered to improve the assay’s precision. Spontaneous release of adenine upon thermal treatment was analyzed by treating samples containing 5 µg/mL ricin or abrin with ssDNA substrate at 85 °C for 60 min. Figure 3 shows the free adenine levels in treated samples and is consistent with the results in Figure 1: both toxins lose most of their activity within 10 min. Trend lines for incubation times >20 min showed a strong correlation between time and peak area, indicating thermal decomposition of ssDNA. Therefore, substrate should be added after cooling, not during heating. To minimize adenine accumulation from thermal degradation and matrix sources, subsequent experiments were performed at 80 °C for 5 min to maximize the differentiation of the two toxins’ activities.

### 2.2. Equation for Calculating Ricin and Abrin Concentrations

After identifying the conditions that maximize the difference in the toxicity between ricin and abrin, two equations are required to calculate the concentrations of both toxins in a mixed sample. If there is a mixed sample of ricin and abrin, their concentrations could be put as A and B. The total toxicity of the untreated sample (T_NT_) and treated sample (T_T_) can be represented as a system of two linear equations in terms of the concentrations of ricin and abrin:

(1) $$T_{NT}=k_{1A}\times A+b_{1A}+k_{1B}\times B+b_{1B}$$

(2) $$T_{T}=k_{2A}\times A+b_{2A}+k_{2B}\times B+b_{2B}$$

And rearranging these equations in terms of each toxin’s concentration yields the following expressions:

(3) $$A=\;\frac{k_{2B}\left(P-b_{1A}-b_{1B}\right)-k_{1B}(Q-b_{2A}-b_{2B})}{k_{1A}k_{2B}-k_{1B}k_{2A}}\;$$

(4) $$B=\frac{k_{1A}\left(Q-b_{2A}-b_{2B}\right)-k_{2A}(P-b_{1A}-b_{1B})}{k_{1A}k_{2B}-k_{1B}k_{2A}}\;$$

where k_1A_/b_1A_ and k_1B_/b_1B_ represent the slope/y-intercept of the standard curve of untreated ricin and abrin, and k_2A_/b_2A_ and k_2B_/b_2B_ represent the slope/y-intercept of the standard curve of the untreated toxins.

**Figure 4:**
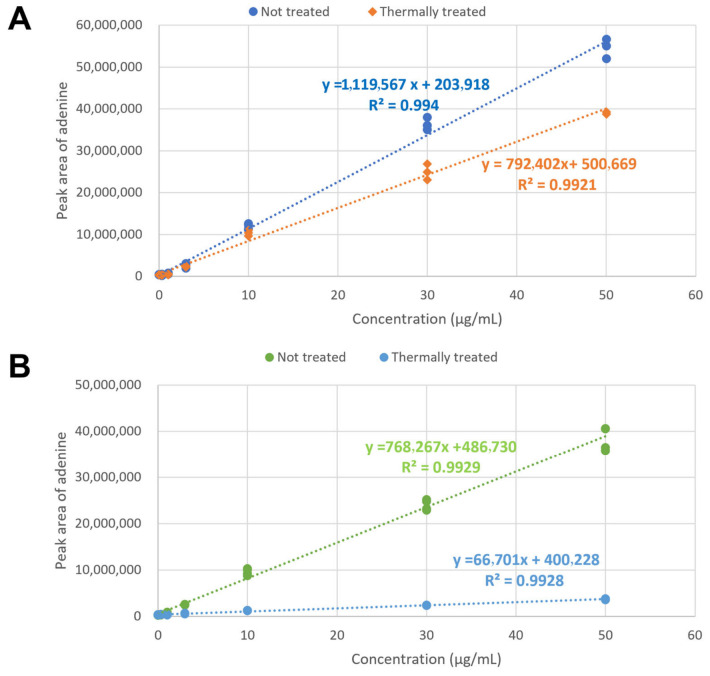
Standard curves for ricin (**A**) and abrin (**B**) toxic activity, ranging from 0.3 to 50 µg/mL, with and without thermal treatment (80 °C, 5 min), as indicated. Dotted lines represent the corresponding standard curves. Each point is an independent replicate from three separate assays.

To solve the equations, four standard curves should be acquired. Ricin and abrin references were prepared at 0.3, 1, 3, 10, 30, and 50 µg/mL, and a standard curve was generated using summed peak areas at 92, 94, and 119 *m*/*z*. Figure 4 is the resulting standard curve. The standard curve for ricin did not change significantly during thermal inactivation, whereas the abrin value decreased by approximately 95%.

**Figure 5:**
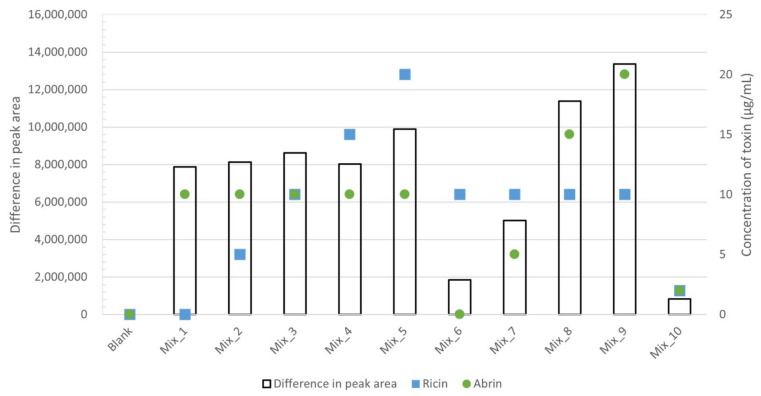
Difference in activity between thermally treated and control samples. The difference was calculated as the average total peak area of control samples minus that of thermally treated samples. The concentrations of ricin and abrin in each sample are indicated in blue and green, respectively.

To validate the equation, 10 samples with varying ricin and abrin concentrations (Table 1) were prepared in sixfold replicates; three replicates per sample were heated to 80 °C for 5 min. After adding ssDNA substrate and performing depurination, adenine peak areas from untreated (T_NT_) and treated (T_T_) samples were used to calculate ricin and abrin concentrations. The calculated values closely matched the spiked concentrations and correctly identified toxin-free samples. However, accuracy decreased at lower concentrations (Mix_10), thereby limiting the method’s lower detection capability. Figure 5 illustrates the difference in peak area between the conditions, overlaid with the spiked concentrations of ricin or abrin. These results indicate that the observed difference in activity primarily originated from the concentration of abrin rather than ricin.

However, the accuracy of the proposed method decreased at lower concentrations (Mix_10), thereby limiting the method’s lower detection capability. Despite these limitations, the calculation results successfully estimated the concentrations of the two toxins.

## 3. Discussion

Although this study used depurination coupled with LC–MS/MS analysis to evaluate toxicity, the proposed approach is not limited to this analytical method. Other methods, such as cell-free translation or equivalent assays, could be readily substituted.

Multiple ribosome-inactivating proteins (RIPs) exist in nature, and co-occurring RIPs may confound depurination measurements and produce false positives. To assess this risk, saporin (a Type I RIP) was tested alongside ricin and abrin. It retained activity up to 75 °C, which is similar to the thermal profile of ricin (Supplementary Data S3). It demonstrated that any contaminating RIPs out of the scope would induce unexpected measurements. Therefore, this method should be complemented by structural identification techniques (e.g., immunoassay or mass spectrometry) and applied only when other RIPs are absent.

Although the method has several limitations, the simultaneous quantification of ricin and abrin in mixtures is otherwise challenging without expensive separation methods or antibodies, and this assay offers practical utility for forensic analysis and bioterrorism attribution.

This study measured only A-chain depurinating activity, whereas overall toxicity depends on both chains. Previous research suggests that the B-chain of abrin degrades upon heating, which may explain its thermal sensitivity [20].

Moreover, this method was evaluated using purified samples without complex matrices, whereas real-world samples contain matrices that may interfere with the treatment. Plasma and skim milk samples were prepared with minimal processing, including centrifugation to remove insoluble material, followed by ultrafiltration to eliminate strong ions in the sample. Differential thermal inactivation was partially observed in milk samples, allowing detection of abrin; however, it was ineffective in plasma samples (Supplementary Data S4). Therefore, more stringent preparation—such as affinity capture using asialofetuin [18] or immobilized galactose—is required to extend the method to real-world samples.

## 4. Conclusions

This study developed a thermal treatment method for the simultaneous quantification of ricin and abrin in mixed samples without the need for antibodies or physical separation. Validation samples showed good agreement between calculated and spiked concentrations. Furthermore, previous studies indicate that the B-chain of RIPs is susceptible to thermal denaturation under the conditions used in this study. Additional research is needed to characterize the thermal stability of the B-chain and to improve the correlation between measured activity and overall toxicity functionality across the intoxication pathway.

## 5. Materials and Methods

### 5.1. Preparation of Ricin and Abrin

Ricin and abrin were extracted from castor beans and rosary peas, respectively. Castor beans were purchased from a local agricultural market without cultivar information. Rosary peas were purchased from Sandeman Seeds (Dublin, Ireland). Peas were ground, extracted with phosphate-buffered saline, and centrifuged to remove lipids and precipitates. Dissolved proteins were purified using a hydroxyapatite column and size exclusion chromatography to remove impurities, including nontoxic agglutinins. Since extracted toxins are highly toxic, safety considerations including personal protective equipment, decontaminating equipment and wastes with 10% bleach solution, and supervision by internal safety committee were applied. Detailed procedures were adapted from previous research [25]. The purity of the extracted toxins was assessed using SDS-PAGE (Supplementary Data S1).

### 5.2. Adenine Depurination Assay

Substrate ssDNA (5′-CGCGCGAGAGCGCG-3′) was synthesized by Bioneer (Daejeon, Republic of Korea) and dissolved in 10 mM Tris, pH 8.0, to a final concentration of 0.1 mM. Depurination protocols were adapted from Becher et al. [11] with modifications. The 10× reaction buffer was 0.5 M ammonium acetate, pH 4.5. Just before depurination, 20 µL of 100 μM ssDNA substrate was added to minimize the spontaneous release of free adenine upon heating. Depurination was performed at 37 °C for 4 h (ThermoMixer, Eppendorf, Hamburg, Germany). Reactions were quenched by adding 1% volume of 0.1 M spermine (Sigma-Aldrich, Seoul, Republic of Korea) to block purine base protonation [26]. Samples were vortexed and centrifuged, and supernatants were transferred to LC vials for LC-MS/MS analysis.

### 5.3. Thermal Inactivation

Samples were mixed with reaction buffer and aliquoted to 0.1–1.5 mL EP tubes for heating in a ThermoMixer. The samples were heated by inserting the tube into the preheated Mixer, then left at room temperature to cool. Substrate ssDNA was added to the tubes as described above for depurination.

### 5.4. LC-MS/MS Analysis of Free Adenine

A Thermo Ultimate 3000 UPLC (Thermo Fisher, San Jose, CA, USA) equipped with Waters CORTECS C18+ (2.1 × 100 mm, 1.6 μm) was used for analysis. The mobile phases consisted of deionized water (Solvent A) or methanol (Solvent B) with 10 mM ammonium formate. Each sample (5 µL) was injected and separated under the following gradient conditions: 0% B from 0 to 2 min, increasing to 50% B from 2 to 3 min, held for 3 to 4 min, then decreased to 0% B from 4 to 5 min, and held for 1 min. The flow rate was 0.2 mL/min. Thermo TSQ Quantiva (Thermo Scientific Korea, Seoul, Republic of Korea) with electrospray ionization was used for MS/MS analysis. Effluents were ionized with positive polarity at 3.5 kV. The precursor ion of *m*/*z* 136 was fragmented to *m*/*z* 92 (25 eV), *m*/*z* 94 (18 eV), and *m*/*z* 119 (25 eV) using the SRM method, and all fragment ions were used for quantitation. The validation result of the analysis method can be found in Supplementary Materials (Supplementary Data S2). Argon was used as a collision gas. XCalibur 3.0.63 (Thermo Fisher, San Jose, CA, USA) was used to control the instruments, and Skyline 25.1 (MacCoss Lab Software, University of Washington, Seattle, WA, USA) was used to process acquired data for peak picking and quantitation.

## 6. Patents

A patent application is pending for the work reported in this manuscript.

## Figures and Tables

**Table 1:** List of mixed samples prepared for validation. Samples were thermally treated for 5 min at 80 °C. Total activity was measured, and toxin concentrations were calculated against the standard curves. Results were derived from three independent assays.

Sample ID	Spiked Ricin (µg/mL)	Spiked Abrin (µg/mL)	Calculated Ricin Concentration * (µg/mL)	Calculated Abrin Concentration * (µg/mL)
Mix_1	0	10	−0.92 ±0.36	11.73 ±2.47
Mix_2	5	10	4.39 ±2.03	9.65 ±1.64
Mix_3	10	10	9.36 ±2.78	8.05 ± 1.41
Mix_4	15	10	15.38 ±4.57	4.46 ±2.07
Mix_5	20	10	18.94 ±5.00	5.45 ±1.87
Mix_6	10	0	8.23 ±2.39	−0.93 ±2.14
Mix_7	10	5	10.60 ±4.62	2.42 ±3.11
Mix_8	10	15	9.75 ±3.03	11.75 ±1.71
Mix_9	10	20	9.14 ±2.79	14.82 ±1.41
Mix_10	2	2	0.89 ±0.64	1.01 ±0.65

* Calculations based on toxic activity.

## Data Availability

The original contributions presented in this study are included in the article/Supplementary Materials. Further inquiries can be directed to the author.

## Supplementary Materials

The following supporting information can be downloaded at: https://www.mdpi.com/article/10.3390/toxins18050233/s1. Supplementary Data S1. SDS-PAGE of purified ricin and abrin; Supplementary Data S2. Validation of LC-MS/MS method for adenine detection; Supplementary Data S3. Thermal stability of saporin over tested treatment conditions in the study; Supplementary Data S4. Thermal stability of ricin/abrin mixed sample in plasma or skim milk.

## Abbreviations

The following abbreviations are used in this manuscript:

SRL	Sarcin/Ricin Loop
OPCW	Organisation for Prohibition of Chemical Weapons
MWCO	Molecular weight cut-off
RIP	Ribosome-inactivating protein
