# Supplementary material for: Differential Thermal Inactivation Enables Simultaneous Quantitation of Ricin and Abrin
Source: Toxins (Basel). 2026 May 19;18(5):233. doi: 10.3390/toxins18050233 (PMC13211329; doi:10.3390/toxins18050233)
Supplement: Supplementary file 1 [file toxins-18-00233-s001.zip › toxins-4239812-supplementary.pdf]

Supplementary Materials: Differential Thermal Inactivation Enables Simultaneous Quantitation of Ricin and Abrin

Woo-Hyeon Jeong

Supplementary data S1. SDS-PAGE of purified ricin and abrin

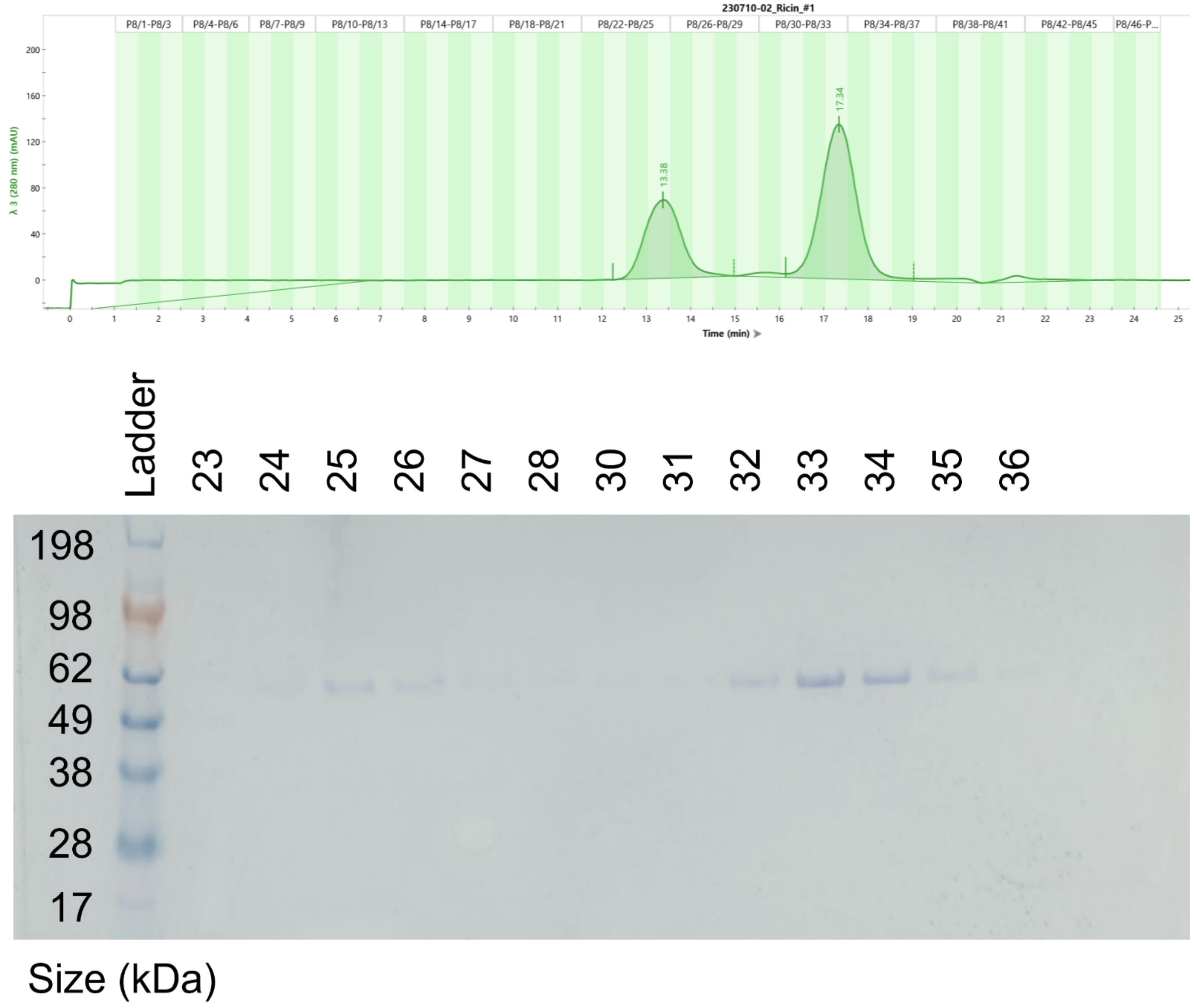

Figure S1. Size exclusion chromatography result (top) and non-reducing SDS-PAGE of each fraction (bottom) of ricin to separate nontoxic agglutinins from toxins. Superdex 200 (Thermo Scientific Korea, Seoul) was used with PBS as running buffer. from 32 to 35 contained ricin.

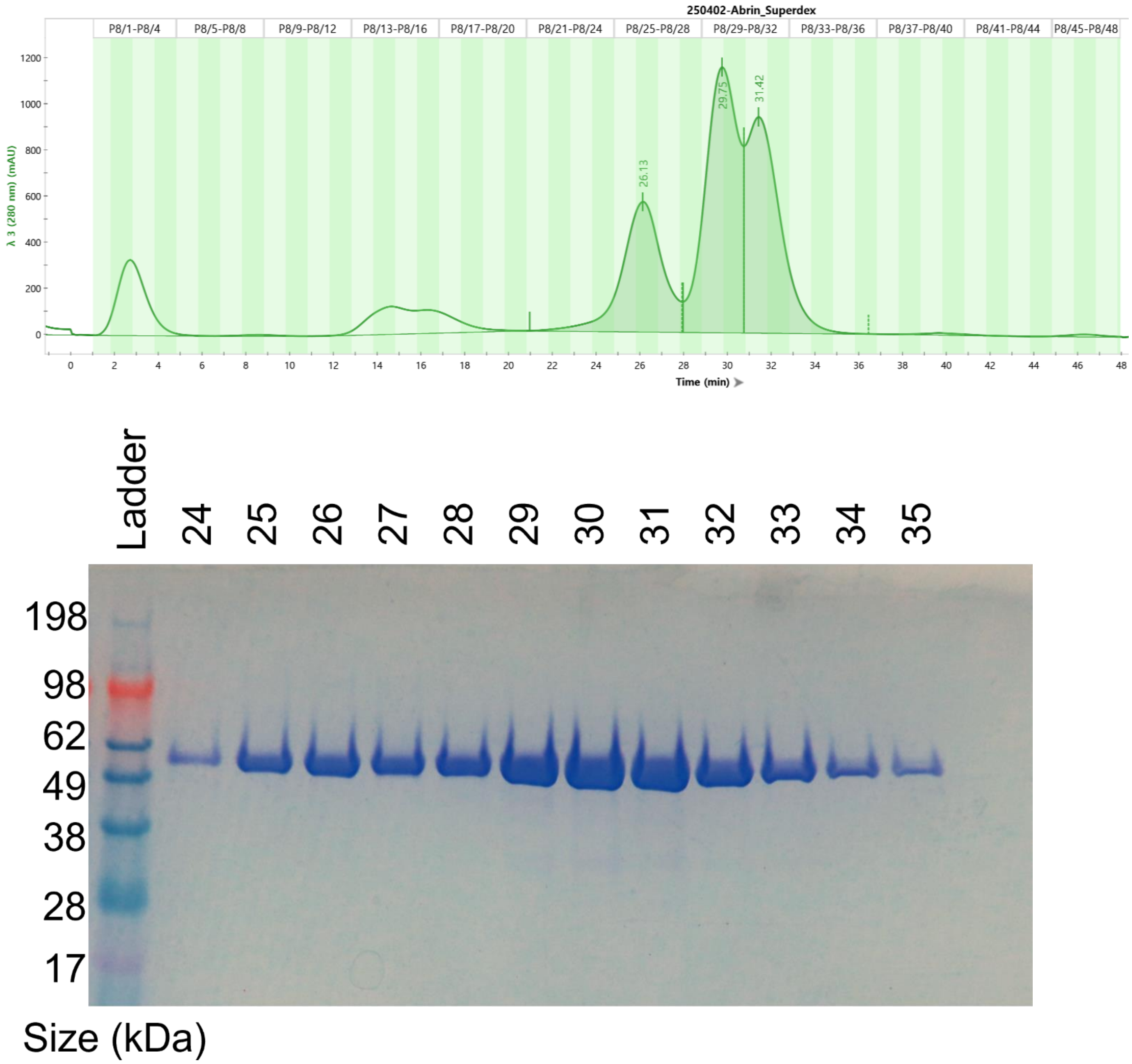

Figure S2. Size exclusion chromatography result (top) and non-reducing SDS-PAGE of each fraction (bottom) of abrin to separate nontoxic agglutinins from toxins. Superdex 200 (Thermo Scientific Korea, Seoul) was used with PBS as running buffer. Fractions from 29 to 33 contained abrin.

Supplementary data S2. Validation of LC-MS/MS method for adenine detection

Table S1. Measurements for standard curve

**Concentration (ng/mL)**	**Peak area** *m/z* **136 → 119**	**Peak area** *m/z* **136 → 94**	**Peak area** *m/z* **136 → 92**	**Summed peak area**
0	9452.0	4331.8	5013.2	18797.11
0.977	31159.6	6466.1	15177.7	52803.39
3.907	99766.9	17337.7	37685.2	154789.7
15.625	645008.3	100545.5	218373.5	963927.3
62.5	2655984.5	397767.1	847780.9	3901532
250	9563200.0	1526993.4	3142309.5	14232503
1000	34223184.0	5631192.5	11370665.0	51225042
0	10483.2	2174.2	5342.8	18000.27
0.977	42304.2	9060.3	21850.9	73215.33
3.907	156081.3	23971.4	52757.3	232809.9
15.625	631166.8	97517.9	209103.4	937788.1
62.5	2612324.0	405582.1	858960.6	3876867
250	9513949.0	1520908.5	3164346.0	14199204
1000	32106666.0	5291752.5	10662957.0	48061376
0	8002.4	2866.4	8187.8	19056.66
0.977	43595.7	7347.4	17969.2	68912.27
3.907	148786.8	25510.2	58832.8	233129.8
15.625	615356.8	93785.8	202342.3	911484.9
62.5	2529423.8	387268.0	820112.4	3736804
250	9249834.0	1475177.1	3066746.0	13791757
1000	31111562.0	5123347.5	10333111.0	46568021

Figure S3. LC-MS/MS analysis result of released adenine by ricin activity.

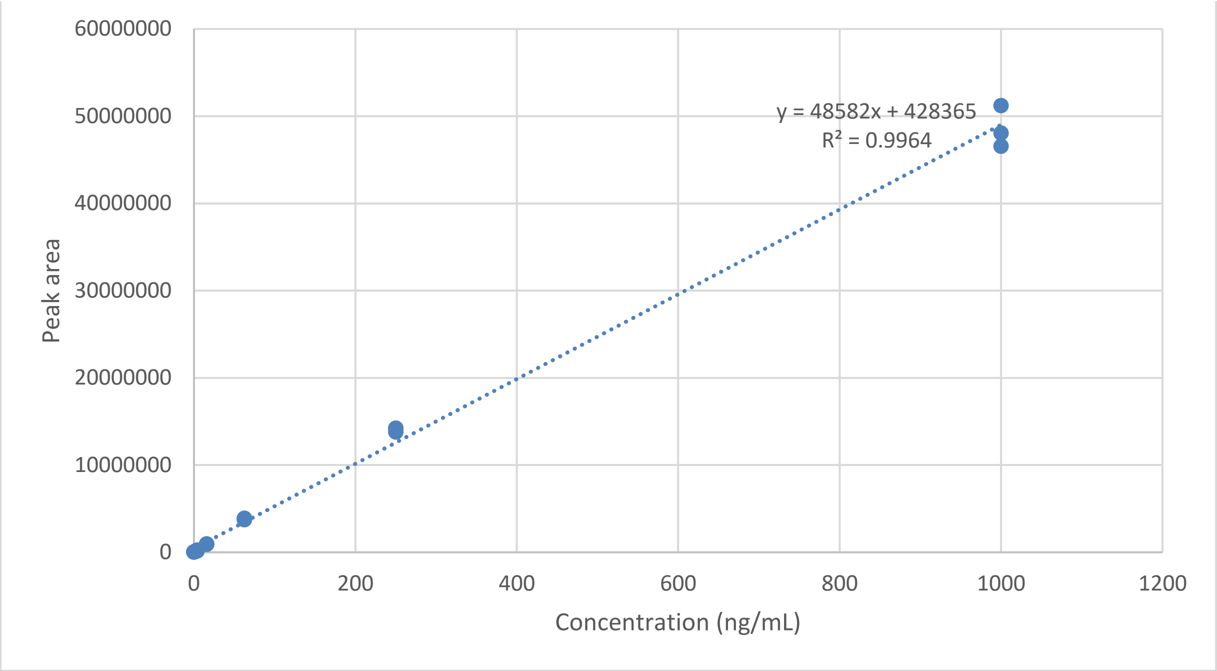

Figure S3. Standard curve and its slope, y-intercept and coefficient of determination.

Table S2. Measurements for calculating standard deviation of the lowest concentration, and the resulting average and standard deviation.

**Concentration (ng/mL)**	**Peak area** m/z **136 → 119**	**Peak area** m/z **136 → 94**	**Peak area** m/z **136 → 92**	**Summed peak area**
0.977	134836.281	20024.734	74124.2578	228985.3
0.977	143833.938	20992.992	60596.6133	225423.5
0.977	174650.172	20513.758	49332.8906	244496.8
0.977	150369.172	20569.459	44194.293	215132.9
0.977	124420.875	17728.684	31826.8945	173976.5
0.977	90147.2734	12967.065	36135.6094	139249.9
0.977	137983.922	21475.934	45874.3906	205334.2
0.977	98583.7344	13647.048	28206.2441	140437
0.977	83145.2813	10230.237	31089.7324	124465.3
0.977	103902.742	18273.973	27397.5664	149574.3
Average	-	-	-	184707.6
Std. deviation	-	-	-	44157.7

$$Limit\ of\ Detection = \frac{3.3\ \times (StDev\ of\ lowest\ concentration)}{(Slope\ of\ the\ standard\ curve)} = \frac{3.3\ \times 44157.7}{48582} = 3.000\ ng/mL$$

Equation S1. Calculation determining limit-of-detection of the method

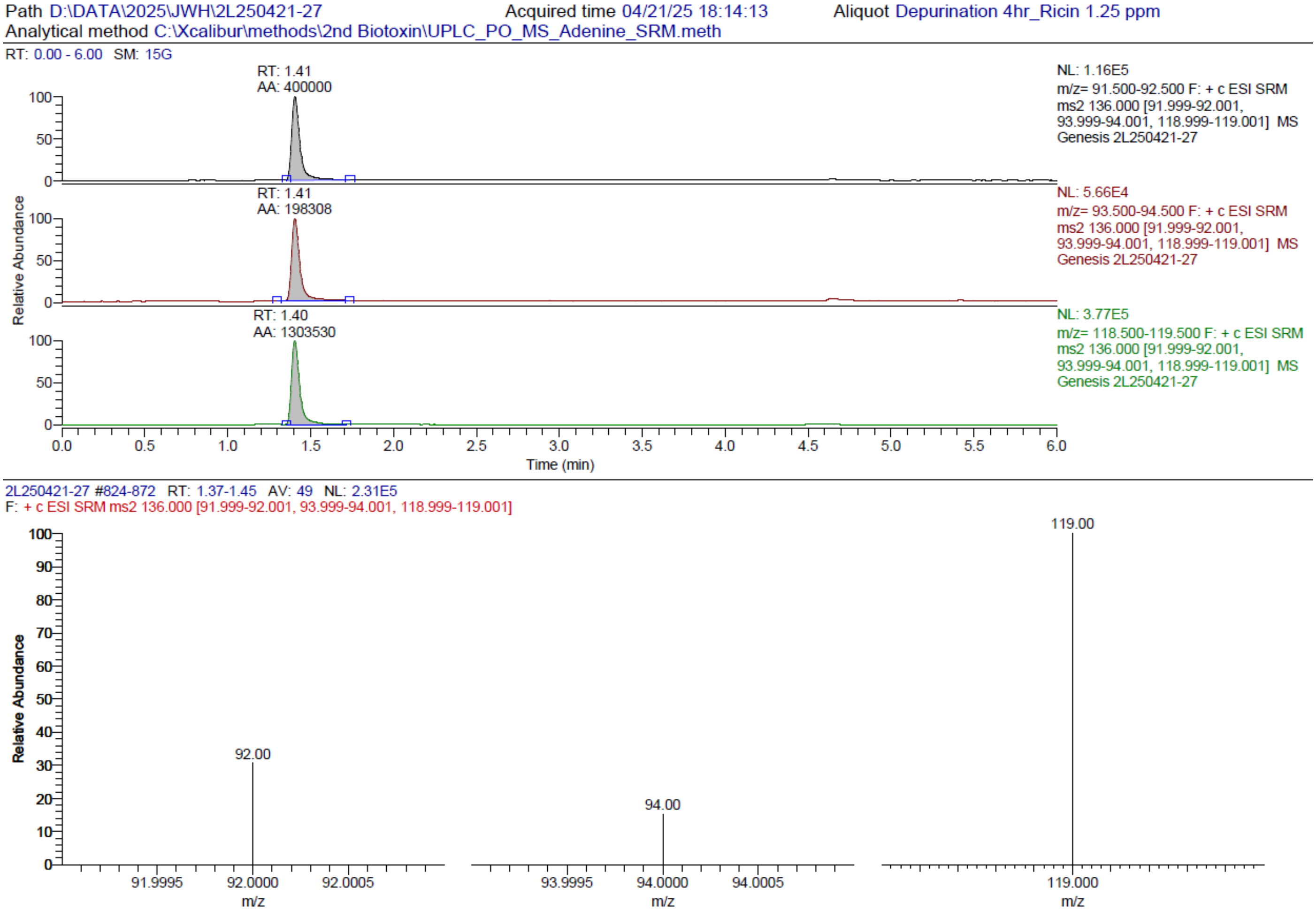

Figure S4. Chromatogram peak of each translation (top) and relative intensities of transitions (bottom) of adenine measured in the method

Supplementary data S3. Thermal stability of saporin over tested treatment conditions in the study

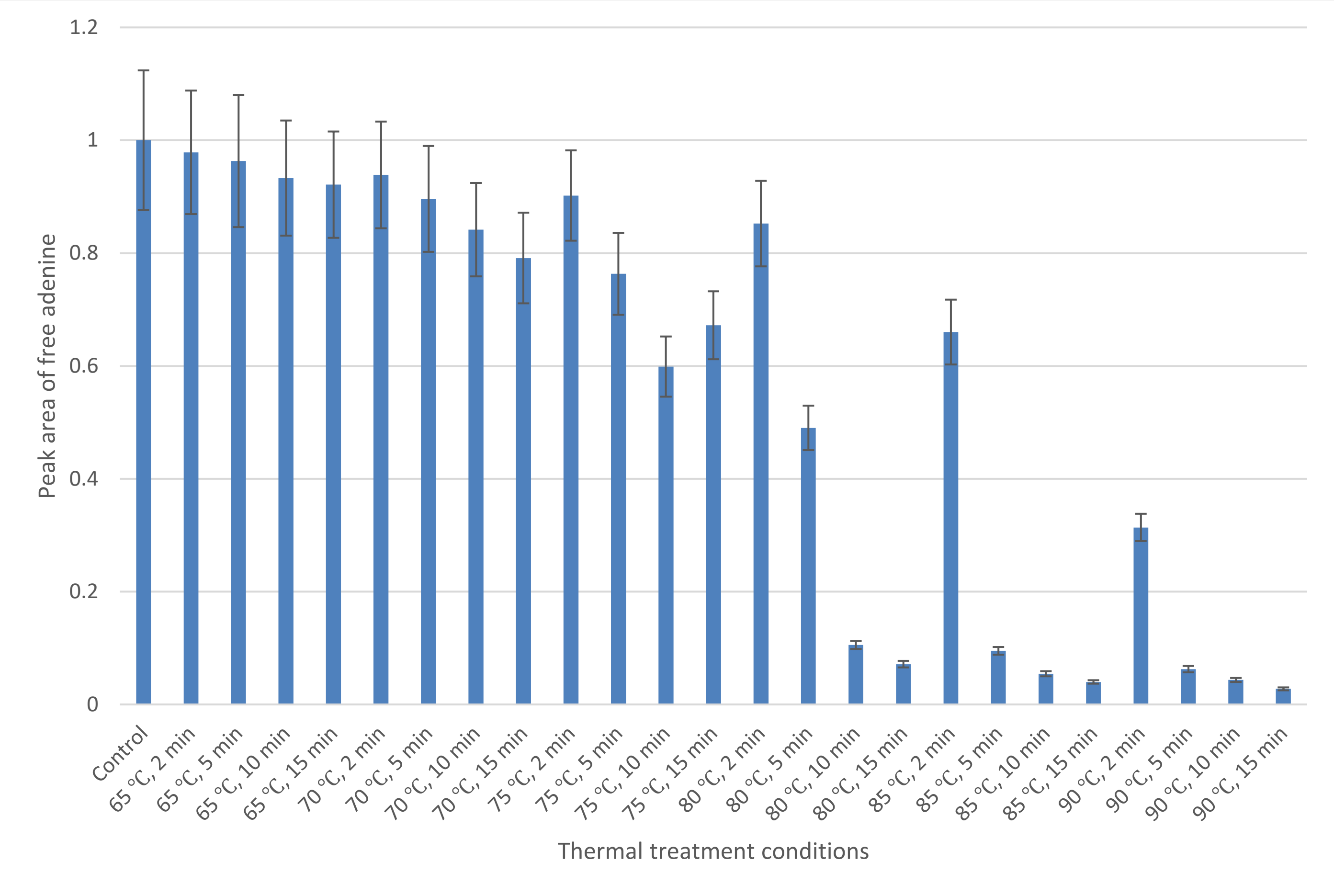

Figure S5. Thermal stability of saporin (Sigma-Aldrich, Seoul, Korea) over tested treatment conditions used in the study. 100 ug/mL of saporin was thermally treated by corresponding conditions, and cooled down to room temperature. To the cooled samples were added substrate ssDNA and incubated at 37 °C for 4 hours. Released free adenine by its toxic activity were measured by LC-ESI-MS/MS. Three independent experiment was performed, and standard deviation was described as error bars.

Supplementary data S4. Thermal stability of ricin/abrin mixed sample in plasma or skim milk

Table S3. List of mixed samples in skim milk (Sigma-Aldrich, Seoul, Korea) prepared for validation. Samples were thermally treated for 5 minutes at 80°C. Total activity was measured, and toxin concentrations were calculated against the standard curves. Results were derived from three independent assays.

Sample ID	Spiked ricin (μg/mL)	Spiked abrin (μg/mL)	Calculated ricin concentration* (μg/mL)	Calculated abrin concentration* (μg/mL)
Milk_blank	0	0	−0.72 ± 0.06	0.59 ± 0.04
Milk_1	0	20	−0.66 ± 0.04	1.13 ± 0.01
Milk_2	10	20	−0.44 ± 0.11	1.33 ± 0.07
Milk_3	20	20	−0.24 ± 0.13	1.86 ± 0.38
Milk_4	30	20	0.07 ± 0.17	1.56 ± 0.31
Milk_5	20	0	−0.19 ± 0.12	0.73 ± 0.18

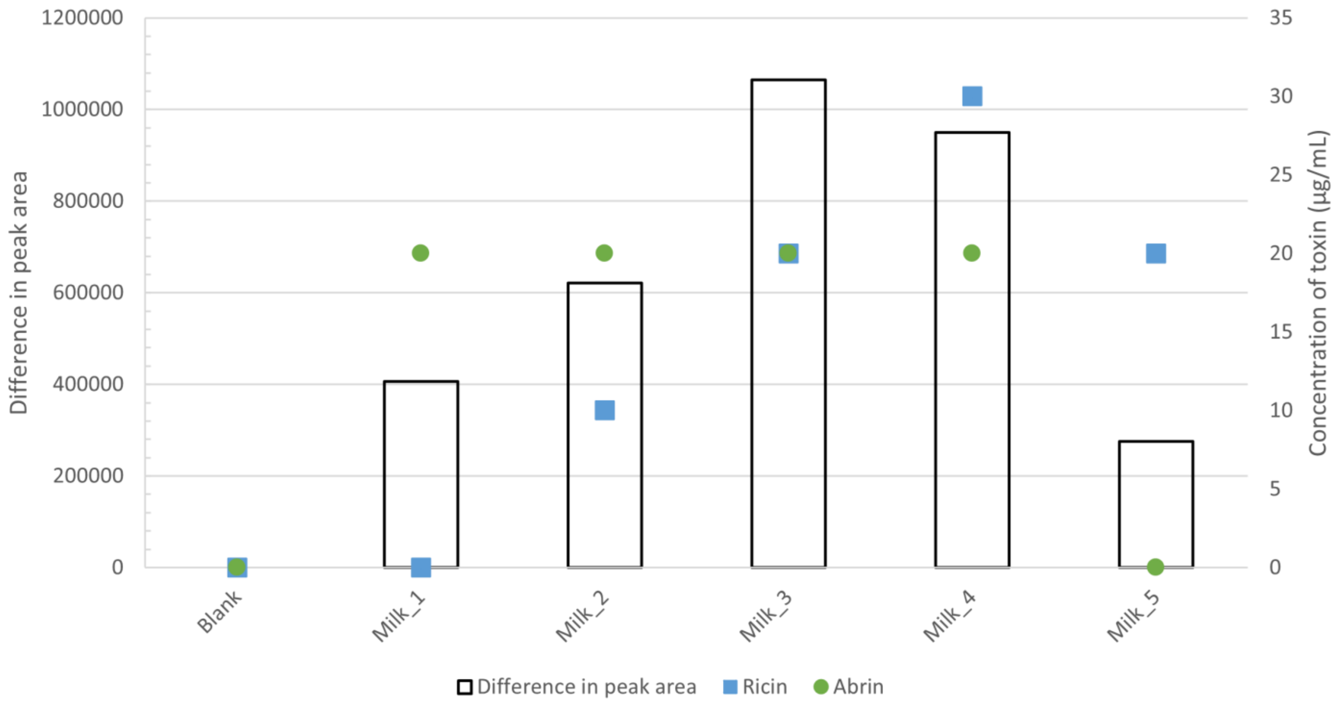

Figure S6. Activity difference of milk-spiked sample between thermally treated one and the control. The difference was calculated by subtracting average of total peak area of thermally treated one from the corresponding value from the control. Concentration of ricin and abrin in each sample was pointed as blue and green, respectively.

Table S4. List of mixed samples in human plasma (HUMANPLK2-0101481, BioIVT, Woodbury, NY) prepared for validation. Samples were thermally treated for 5 minutes at 80°C. Total activity was measured, and toxin concentrations were calculated against the standard curves. Results were derived from three independent assays.

Sample ID	Spiked ricin (μg/mL)	Spiked abrin (μg/mL)	Calculated ricin concentration* (μg/mL)	Calculated abrin concentration* (μg/mL)
Plasma_blank	0	0	−1.19 ± 0.02	1.38 ± 0.42
Plasma _1	0	20	−0.62 ± 0.08	0.43 ± 0.19
Plasma _2	10	20	−0.62 ± 0.13	0.40 ± 0.23
Plasma _3	20	20	−0.60 ± 0.01	0.36 ± 0.07
Plasma _4	30	20	−0.66 ± 0.07	0.43 ± 0.17
Plasma _5	20	0	−0.71 ± 0.07	0.55 ± 0.26

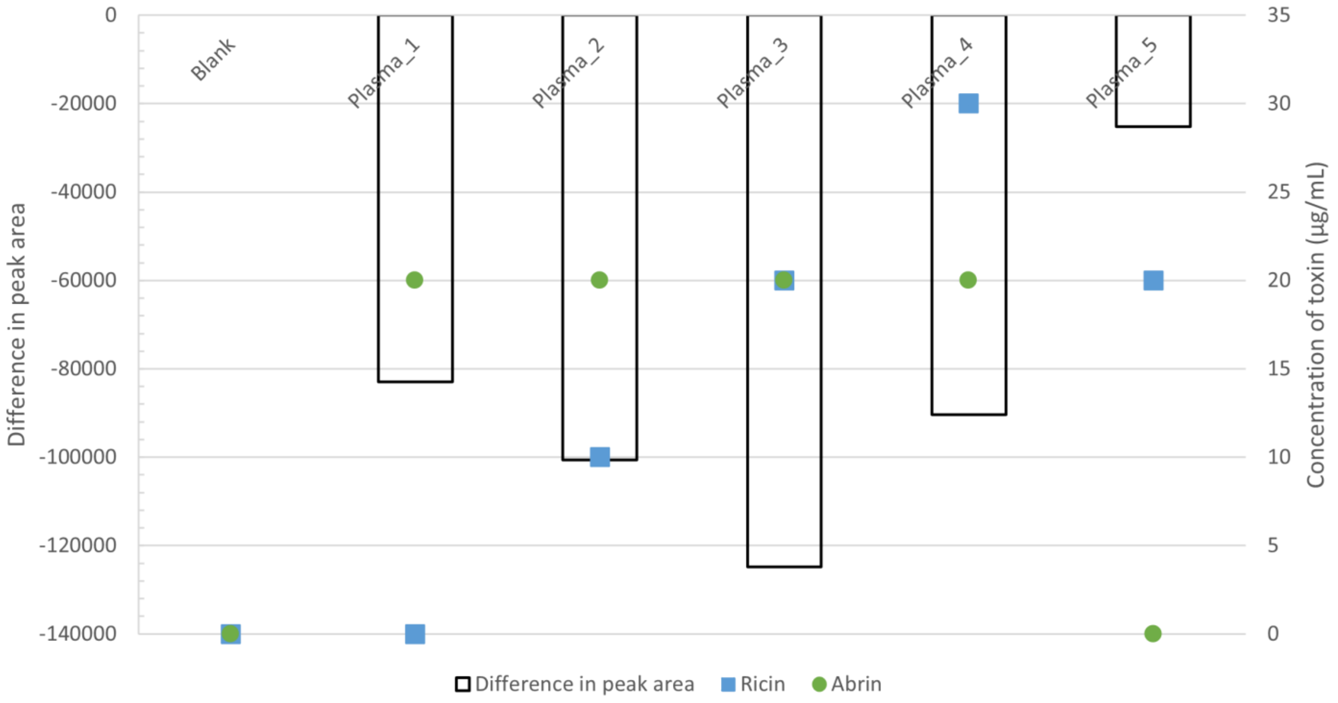

Figure S7. Activity difference of plasma-spiked sample between thermally treated one and the control. The difference was calculated by subtracting average of total peak area of thermally treated one from the corresponding value from the control. Concentration of ricin and abrin in each sample was pointed as blue and green, respectively.
